# Genome-Wide Analysis in Three *Fusarium* Pathogens Identifies Rapidly Evolving Chromosomes and Genes Associated with Pathogenicity

**DOI:** 10.1093/gbe/evv092

**Published:** 2015-05-19

**Authors:** Jana Sperschneider, Donald M. Gardiner, Louise F. Thatcher, Rebecca Lyons, Karam B. Singh, John M. Manners, Jennifer M. Taylor

**Affiliations:** ^1^CSIRO Agriculture Flagship, Centre for Environment and Life Sciences, Perth, Western Australia, Australia; ^2^CSIRO Agriculture Flagship, Queensland Bioscience Precinct, Brisbane, Queensland, Australia; ^3^University of Western Australia Institute of Agriculture, University of Western Australia, Crawley, Western Australia, Australia; ^4^CSIRO Agriculture Flagship, Black Mountain Laboratories, Canberra, Australian Capital Territory, Australia

**Keywords:** *Fusarium*, fungal pathogens, diversifying selection, effector, dispensable chromosomes, evolution

## Abstract

Pathogens and hosts are in an ongoing arms race and genes involved in host–pathogen interactions are likely to undergo diversifying selection. *Fusarium* plant pathogens have evolved diverse infection strategies, but how they interact with their hosts in the biotrophic infection stage remains puzzling. To address this, we analyzed the genomes of three *Fusarium* plant pathogens for genes that are under diversifying selection. We found a two-speed genome structure both on the chromosome and gene group level. Diversifying selection acts strongly on the dispensable chromosomes in *Fusarium oxysporum* f. sp. *lycopersici* and on distinct core chromosome regions in *Fusarium graminearum*, all of which have associations with virulence. Members of two gene groups evolve rapidly, namely those that encode proteins with an N-terminal [SG]-P-C-[KR]-P sequence motif and proteins that are conserved predominantly in pathogens. Specifically, 29 *F. graminearum* genes are rapidly evolving, in planta induced and encode secreted proteins, strongly pointing toward effector function. In summary, diversifying selection in *Fusarium* is strongly reflected as genomic footprints and can be used to predict a small gene set likely to be involved in host–pathogen interactions for experimental verification.

## Introduction

Numerous genes in fungi have been shown to undergo diversifying selection, in particular those linked to environmental adaptation, niche specialization, and host–pathogen interactions (Gladieux et al. 2014). Host and pathogen apply strong selective pressure on each other and over time this coevolutionary process leaves genomic footprints. Adaptive evolution can either be measured on the short time-scale of population variation or the long time-scale of divergent species (Kryazhimskiy and Plotkin 2008). A short time-scale analysis using polymorphism data is powerful for investigating recent events such as emerged diseases or population migration dynamics. On the other hand, a long time-scale analysis using divergence data is able to elucidate forces in long-term coevolutionary dynamics such as the host–pathogen arms race and possibly processes of host specialization. Here, the ratio of nonsynonymous to synonymous substitutions $d_{N}/d_{S}$ in orthologous genes of related species is used to assess diversifying selection.

In filamentous plant pathogens, diversifying selection has been shown to act particularly on genes that encode effector proteins (Aguileta et al. 2009; Dodds 2010). Effectors are active outside the fungal cell and interfere with host defenses including pathogen recognition and signaling processes mediated by the host. Most studies have focused on investigating diversifying selection in effectors such as host-specific toxins, avirulence (*Avr*) genes, and elicitors on a case-by-case basis. For example, adaptive evolution was found in the mature protein region of the phytotoxin-like *scr74* gene family of the oomycete *Phytophthora infestans* (Liu et al. 2005), in the C-terminal of *Phytophthora* RXLR effector paralogs (Win et al. 2007) and a single amino acid polymorphism in the *Phytophthora* EPIC1 effector has been linked to the ability of specializing on a new host (Dong et al. 2014). In fungal pathogens, polymorphism data have revealed two codons under diversifying selection in the host-specific necrotrophic effector *ToxA*, produced by the wheat pathogens *Pyrenophora tritici-repentis* and *Stagonospora nodorum* (Stukenbrock and McDonald 2007). Host adaptation or host evasion processes were suggested for six genes encoding cell wall degrading enzymes in *Zymoseptoria tritici* (Brunner et al. 2013). Plant resistance (*R*) genes control recognition of pathogens carrying specific *Avr* effectors in a gene-for-gene manner and the *AvrL567*, *AvrP123*, and *AvrP4* genes have been shown to be highly polymorphic in the rust pathogen *Melampsora lini* in a suspected arms race with the host plant (Dodds et al. 2006; Barrett et al. 2009). Next-generation sequencing technologies have delivered a large number of available pathogen genomes and have thus made it possible to find signatures of diversifying selection in lineages on a genome-wide level. For example, the fungal wheat pathogen *Z. tritici* shows different patterns of nucleotide evolution on its core and dispensable chromosomes (Stukenbrock et al. 2010). Accelerated rates of evolution were found in gene-sparse regions of *Phytophthora infestans* following host jumps (Raffaele et al. 2010) and in candidate effector genes of the wheat powdery mildew *Blumeria graminis* f. sp. *tritici* (Wicker et al. 2013). A genome-wide study of diversifying selection in the wheat stem rust fungus *Puccinia graminis* f. sp. *tritici* revealed that adaptation occurs predominantly in pathogen-associated gene families and that this signal can be exploited to point to effector candidates (Sperschneider et al. 2014).

The *Fusarium* genus contains filamentous ascomycete fungi that are able to infect a diverse range of plants and cause destructive losses in many economically important food crops. *Fusarium* pathogens have over time considerably diverged in terms of their life cycles, niche specialization, and ability to cause disease on different hosts. Members of the *Fusarium graminearum* species complex cause *Fusarium* head blight or scab of wheat and barley and result in devastating agricultural losses worldwide due to yield reduction as well as their ability to contaminate grains with trichothecene mycotoxins (Goswami and Kistler 2004; Kazan et al. 2012). The *Fusarium oxysporum* species complex has a remarkably broad host range at the species level, causing vascular wilt disease in hundreds of different host species through different formae speciales (Michielse and Rep 2009). Members of the *Fusarium fujikuroi* species complex cause a variety of disease on hosts such as rice, maize, mango, and pine, and the *Fusarium solani* species complex contains members that can infect an astonishingly wide variety of hosts. The evolutionary processes and specialization over time in the genus is likely to be reflected as genomic signatures. Thus far, comparative genome analyses of *Fusarium* chromosome assemblies have suggested a compartmentalization into core and adaptive regions (Ma et al. 2013). In *F. solani* f. sp. *pisi*, the three dispensable chromosomes have been linked to habitat specialization (Coleman et al. 2009) and pathogenicity toward pea (Han et al. 2001). The *F. oxysporum* f. sp. *lycopersici* dispensable chromosome 14 is referred to as a pathogenicity chromosome and has been shown to turn a nonvirulent recipient strain virulent toward tomato via acquisition of the entire chromosome (Ma et al. 2010). In *F. graminearum*, regions of high single nucleotide polymorphism (SNP) density determined by mapping between two strains were found at the ends of chromosomes and additionally in interstitial regions on three of the four chromosomes (Cuomo et al. 2007). However, the full impact of diversifying selection and particularly host adaptation and associated pathogen coevolution on genomic regions as well as on specialized gene groups remains elusive.

Despite the availability of sequenced *Fusarium* genomes and gene expression data, our knowledge of *Fusarium* genes involved in causing disease is fragmentary. Most *Fusarium* pathogens are classified as hemibiotrophs that employ a biotrophic phase initially and later feed on dead host cells to obtain nutrients from them (Thatcher et al. 2009; Ma et al. 2013). Several general pathogenicity factors have been found in *Fusarium* that are also part of conserved complexes or pathways, such as mitogen-activated protein kinases (Ma et al. 2013). On the other hand, specialized *Fusarium* genes involved in host–pathogen interactions such as host-specific toxins, elicitors, or *Avr* genes are largely undefined, apart from the secreted in xylem (SIX) effectors in *F. oxysporum* f. sp. *lycopersici* (Houterman et al. 2007) and several *Fusarium* mycotoxins. However, there is increasing evidence that specialized virulence factors such as bona fide effectors are more common in *Fusarium* than previously thought (Brown et al. 2012; Kazan et al. 2012; Ma et al. 2013).

In this work, we investigated the genome-wide impact of diversifying selection on three *Fusarium* pathogens to answer the following questions: 1) How is the two-speed *Fusarium* genome structure compartmentalized for *Fusarium* species with and without dispensable chromosomes? 2) Is the two-speed genome also reflected on the gene group level and which functional groupings of *Fusarium* genes evolve faster than the remainder of the genome? and 3) Can the signal of adaptation combined with in planta expression data be used to predict genes involved in host-*Fusarium* interactions, such as *Avr* genes, elicitors, and host-specific toxins employed in the early biotrophic phase?

## Materials and Methods

### Genome-Wide Diversifying Selection Analysis

For each protein in *F. graminearum*, *F. oxysporum* f. sp. *lycopersici*, and *F. verticillioides*, phmmer (Finn et al. 2011) was run against the collected *Fusarium* proteomes (table 1), and all significant protein hits (E-value < E-5) and per-domain hits per protein were recorded. Significant protein hits were kept if the combined domain hits for query and target cover more than 60% of the sequences, respectively. This ensured that for the subsequent diversifying selection analysis only reliable and well-conserved multiple alignments were used. Protein multiple sequence alignments were inferred using PRANK with the +F option (Loytynoja and Goldman 2005). Alignment columns with more than 70% gap characters were masked using a custom Python script for preparation of phylogenetic tree prediction. Phylogenetic trees were calculated using the Phyml package version 20120412 (Guindon et al. 2010). Trees were midpoint rooted and orthologs were derived with the species overlap method using ETE (Huerta-Cepas et al. 2010). Each ortholog set was aligned, gaps were masked, and phylogenetic trees were predicted as described above. The gaps in the ortholog protein alignments were used to produce a coding sequence alignment using PAL2NAL (Suyama et al. 2006).

**Table 1:** The Set of *Fusarium* Genomes Used for the Prediction of Diversifying Selection with Their Genome Characteristics

**Species**	**Strain**	**Genome Size (Mb)**	**No. of Chromosomes in Assembly**	**References**
*F. graminearum*	PH-1	36	4	Cuomo et al. (2007)
*F. culmorum*	CS7071	37	Unknown	Unpublished, Genbank accession CBMH010000000
*F. pseudograminearum*	CS3220	37	Unknown	Moolhuijzen et al. (2013)
*F. acuminatum*	CS5907	43	Unknown	Moolhuijzen et al. (2013)
*F. incarnatum*–F. equiseti	CS3069	38	Unknown	Moolhuijzen et al. (2013)
*F. verticillioides*	7600	42	11	Ma et al. (2010)
*F. fujikuroi*	IMI58289	44	12	Wiemann et al. (2013)
*F. oxysporum* f. sp. *lycopersici*	4287	61	15	Ma et al. (2010)
*F. oxysporum*	Fo5176	55	Unknown	Thatcher et al. (2012)

We applied two methods for estimating synonymous and nonsynonymous substitution rates and detection of site-specific diversifying selection, namely YN00 and CODEML from the PAML software (Yang 2007). YN00 was used to calculate pairwise $d_{N}/d_{S}$ ratios and was applied to all genes that have at least one ortholog. If a gene has at least two orthologs, the mean of pairwise $d_{N}/d_{S}$ ratios was calculated and site-specific diversifying selection was additionally assessed using CODEML of the PAML software version 4.7 (Yang 1997), with alignment gaps removed. Two likelihood ratio tests (LRTs) of site-specific diversifying selection were used: model M1 (neutral) to model M2 (selection), and model M7 (beta) to M8 (beta&ω), and significance was assessed with the chi-square tests at the significance threshold *P* < 0.05. A gene was considered to be undergoing site-specific diversifying selection if both the M1/M2 and M7/M8 LRTs were significant. We compared the number of genes that are under site-specific diversifying selection in a particular group or on a chromosome to the remaining gene set of the whole genome. Statistical significance was compared using a Fisher’s exact test at a significance threshold of *P* < 0.05.

### Functional Annotation and Visualization

Pfam was run as a batch search with an E-value threshold of 0.00001 (Finn et al. 2014). MEME was run with the options –nmotifs 30, –minw 5, and –maxw 50 (Bailey et al. 2009). The MIPS FunCat tool was run online with default parameters and Bonferroni corrected *P*-values were reported at significance threshold *P* < 0.05 (Ruepp et al. 2004). Genomic plots were produced with Circos and links between orthologs were bundled using the bundlelinks utility from the Circos plot tools package with parameters set to –strict -max_gap 1e6 -min_bundle_size 2e5 (Krzywinski et al. 2009).

### Fusarium Gene Group Classifications and Analysis of Fusarium [SG]-P-C-[KR]-P Proteins

We used functional classifications of genes encoding carbohydrate-active enzymes and transcription factors given in Ma et al. (2010). We predicted secreted proteins using SignalP 4.1 (Petersen et al. 2011) and the subset of secreted proteins with a small size (<300 amino acids) and more than four cysteines. We collected the set of proteins with an N-terminal [SG]-P-C-[KR]-P sequence motif in the first 50 amino acids of their sequence with a custom Python script and used the set of proteins that can be associated with pathogenicity based on their conservation across the fungal kingdom as given in Sperschneider et al. (2013) for *F. graminearum* and *F. oxysporum* f. sp. *lycopersici*. We obtained the C-terminal protein sequences of the [SG]-P-C-[KR]-P proteins by sliding a window of size 30 amino acids across the sequences until the serine/threonine content in the window was less than 25%. We then manually inspected the C-terminal sequences and made minor adjustments where necessary. To cluster the protein sequences into families, we used TRIBE-MCL (Enright et al. 2002) with phmmer (Finn et al. 2011) all-versus-all scores on the C-terminal sequences.

### In planta Expression Data for F. graminearum

We downloaded publicly available expression data for *F. graminearum* from several experiments: 1) 431 probe sets that were detected exclusively in planta in infected barley (Guldener et al. 2006); 2) 344 genes that were preferentially expressed during invasive growth in planta upon infection of wheat (Zhang et al. 2012) as well as the corresponding Robust Multi-array Average (RMA) treatment means given in PLEXdb experiment FG19 (Dash et al. 2012). Zhang et al. (2012) identified three distinct time points of *F. graminearum* infection in their experiment: 16 HAI (termed covert penetration); 40 HAI (“rapid proliferation”); and 64 HAI (“overt destruction”). The list of 344 of in planta differentially expressed genes was identified by Zhang et al. (2012) as genes that were more strongly expressed during the in planta growth stages than in spores (0 HAI) and hyphae grown in vitro (72 h) as well as compared with spores grown in vitro using previously published data (Seong et al. 2008). For increased sensitivity in detecting genes expressed in planta, we calculated the set of probe sets that have a fold change of greater than 1.5 when comparing in planta infection at a given time point (16, 40 and 64 HAI) to both spores (0 HAI) and hyphae (72 h) using simple two-class comparisons of the RMA expression summaries.

## Results

### Genome-Wide Analysis of Diversifying Selection in the Fusarium Genus

**Fig. 1.—:**
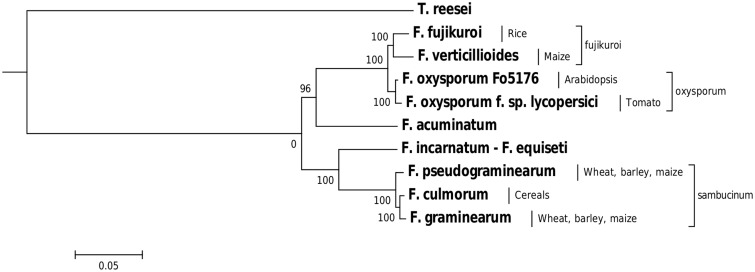
Phylogenetic relationships of the *Fusarium* genomes used for the prediction of diversifying selection with species complex information and host ranges. Nine *Fusarium* genomes were used for the prediction of diversifying selection in the genus. One hundred genes were randomly chosen that have predicted one-to-one orthologies amongst all Fusarium genomes and a phmmer hit with E-value of zero in *Trichoderma reesei* (Martinez et al. 2008). Protein sequences were concatenated and a multiple sequence alignment was calculated using PRANK with the +F option (Loytynoja and Goldman 2005). The tree was constructed using Phyml with branch support values shown (Guindon et al. 2010). *Trichoderma reesei* was used as an outgroup to root the phylogenetic tree.

To identify processes under strong evolutionary pressure in the *Fusarium* genus, we performed a genome-wide analysis of diversifying selection for three reference *Fusarium* genomes: *F. graminearum*, *F. oxysporum* f. sp. *lycopersici*, and *Fusarium verticillioides*, using orthologs from a set of nine *Fusarium* genomes (table 1). *F. graminearum*, *F. oxysporum* f. sp. *lycopersici*, and *F. verticillioides* were chosen because of their distribution across different species complexes, the availability of chromosomal data and the maturity of their gene models and annotations. The phylogenetic relationships and variation in host specificity are shown in figure 1.

For each gene in *F. graminearum*, *F. oxysporum* f. sp. *lycopersici*, and *F. verticillioides*, orthologs in the *Fusarium* genus were predicted using phylogenetic trees and were analyzed for signatures of diversifying selection using two methods from the PAML software (Yang 2007). YN00 uses the counting method of Yang and Nielsen (2000) to estimate pairwise $d_{N}/d_{S}$ ratios and was applied to all genes that have at least one ortholog. If a gene has at least two orthologs, we were able to additionally analyze it for patterns of site-specific diversifying selection using two LRTs of CODEML. This enabled us to calculate $d_{N}/d_{S}$ ratios for 85.8% of genes in *F. graminearum*, 78.5% of genes in *F. oxysporum* f. sp. *lycopersici*, and 85.9% of genes in *F. verticillioides* and site-specific diversifying selection for 83.2% of genes in *F. graminearum*, 69.7% of genes in *F. oxysporum* f. sp. *lycopersici*, and 80.8% of genes in *F. verticillioides* (Results given in supplementary material, Supplementary Material online). The percentage of genes that could be analyzed for diversifying selection for *F. oxysporum* f. sp. *lycopersici* is relatively low due to the number of lineage-specific genes that do not have predicted orthologs in the *Fusarium* genus and the mean $d_{N}/d_{S}$ ratio for the *F. oxysporum* f. sp. *lycopersici* genome is slightly higher at 0.2 than for *F. graminearum* and *F. verticillioides* with 0.15.

### Two-Speed Genome Structure for Fusarium Pathogens with and without Dispensable Chromosomes

**Fig. 2.—:**
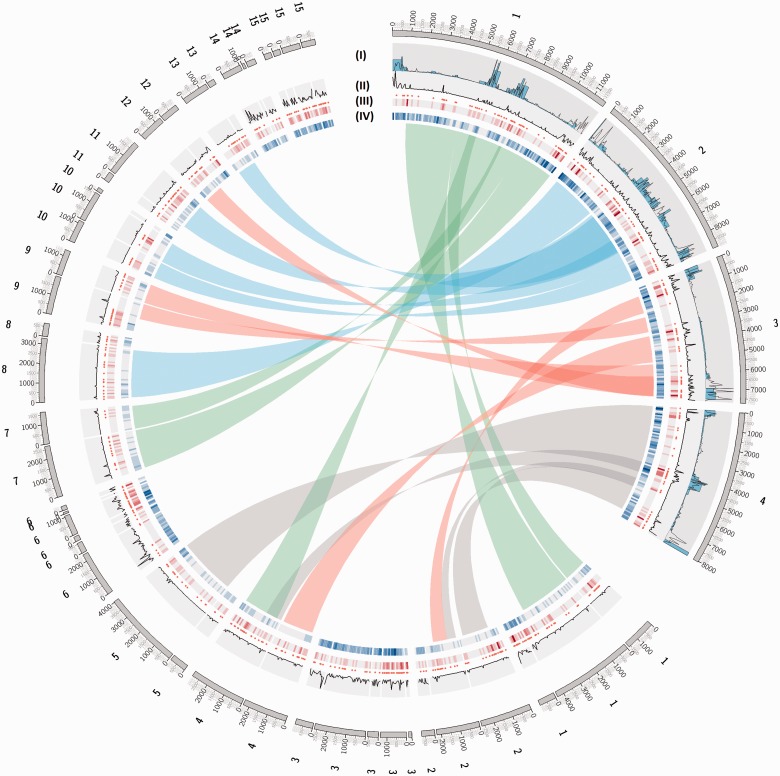
Distribution of genes under diversifying selection across the *F. graminearum* and *F. oxysporum* f. sp. *lycopersici* genomes and regions of macrosynteny based on predicted orthologies. A Circos plot is shown which visualizes the four *F. graminearum* chromosomes followed by the 15 chromosomes of *F. oxysporum* f. sp. *lycopersici* displayed as merged supercontigs with ticks in kilobase (kb) units. The following bands are visualized: (I) Recombination frequency (blue bars) and SNP density (line) for *F. graminearum* (Cuomo et al. 2007), (II) line plot of $d_{N}/d_{S}$ ratios in 50 kb bins, (III) scatter plot and heat map of genes with more than two orthologs that are predicted to undergo site specific diversifying selection using CODEML, and (IV) heat map that visualizes regions for which no ortholog information is available in dark blue. Bundled links for all orthologs between *F. graminearum* and *F. oxysporum* f. sp. *lycopersici* are shown as ribbons, colored according to their chromosomal origin in *F. graminearum*. Note that $d_{N}/d_{S}$ ratios for *F. graminearum* and *F. oxysporum* f. sp. *lycopersici* are not shown on the same scale for clarity.

One of our aims was to identify chromosomal regions in *F. graminearum*, *F. oxysporum* f. sp. *lycopersici*, and *F. verticillioides* that are under diversifying selection in the genus and might point to regions involved in host–pathogen coevolution. We first plotted $d_{N}/d_{S}$ ratios as well as links between orthologs for *F. graminearum* and *F. oxysporum* f. sp. *lycopersici* (fig. 2, track II). This revealed known regions of macrosynteny, which have been attributed to ancient chromosome fusion events (Cuomo et al. 2007; Ma et al. 2010). As expected, regions without predicted orthologies in *F. oxysporum* f. sp. *lycopersici* are found most strikingly on the dispensable chromosomes 3, 6, 14, and 15 as well as at the lineage-specific ends of chromosomes 1 and 2 (fig. 2, track IV). In *F. graminearum*, regions without predicted orthologies occur predominantly in the subtelomeric regions of the chromosomes as well as in the center of chromosome 4. In *F. verticillioides*, regions without predicted orthologies are found in the subtelomeric regions particularly of chromosome 1, 4, 6, and 10 as well as in a small region of around 100 kb in the center of chromosome 7 (supplementary fig. S1, Supplementary Material online, track III).

**Fig. 3.—:**
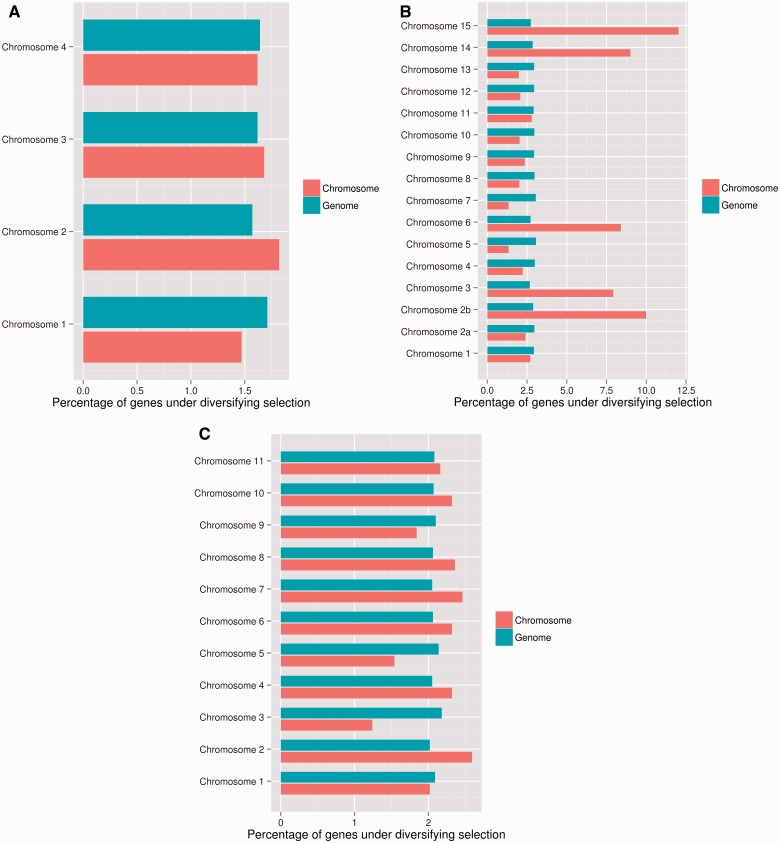
A comparison of diversifying selection acting on the *F. graminearum*, *F. oxysporum* f. sp. *lycopersici*, and *F. verticillioides* chromosomes. Percentages of genes under site-specific diversifying selection for specific chromosomes are shown in red. These were compared with the percentages of genes under site-specific diversifying selection for the remaining chromosomes, shown in blue. Statistical significance for each group compared with the remainder of the genome was assessed with a Fisher’s exact test at a significance threshold of *P* < 0.05. (*A*) *Fusarium graminearum* chromosome 2 has the highest percentage of rapidly evolving genes. (*B*) For *F. oxysporum* f. sp. *lycopersici*, significantly higher numbers of rapidly evolving genes are found on the dispensable chromosomes 3, 6, 14, and 15 as well as chromosomes 2b. (*C*) *F. verticillioides* chromosome 2 has the highest percentage of rapidly evolving genes.

We used statistical testing to determine the significance of differences in the number of genes under site-specific diversifying selection for the individual chromosomes in comparison to the remainder of the genome. On the dispensable *F. oxysporum* f. sp. *lycopersici* chromosomes, the percentage of genes that could be analyzed for site-specific diversifying selection is lower than for the core chromosomes (∼30–40% on dispensable chromosomes have a CODEML result compared with 69.7% genome-wide). Nevertheless, *F. oxysporum* f. sp. *lycopersici* has a significantly higher percentage of rapidly evolving genes on the dispensable chromosomes 3, 6, 14, and 15 as well as on chromosomes 2b (fig. 3*B*). Interestingly, the highest percentage of rapidly evolving genes for *F. graminearum* is found on chromosome 2 (fig. *3**A*). For *F. verticillioides* high percentages of rapidly evolving genes are located on chromosome 2, 7, 8, 6, 10, and 4 (fig. 3*C*).

For *F. graminearum*, we find that SNP density (Cuomo et al. 2007) is positively correlated with the distribution of $d_{N}/d_{S}$ ratios for the same window size of 50 kb (fig. 2, tracks I and II). However, we additionally identify an interstitial region on chromosome 3 that contains a high number of genes that are under site-specific diversifying selection. It is possible that this region does not show a high number of polymorphic residues when using population data, but has nevertheless undergone adaptation in independent lineages, possibly linked to ancient host or niche specialization*.* For *F. oxysporum* f. sp*. lycopersici* the distribution of $d_{N}/d_{S}$ ratios in 50-kb windows shows peaks on the dispensable chromosomes 3, 6, 14, and 15 as well as at the ends of chromosomes 1 and 2 (fig. 2, track II). For *F. verticillioides*, the distribution of $d_{N}/d_{S}$ ratios in 50-kb windows has peaks on chromosomes 8, 10, and 11 as well as in subtelomeric regions of several other chromosomes (supplementary fig. S1, Supplementary Material online, track II).

A MIPS FunCat functional enrichment analysis of *F. graminearum* genes reveals that, unlike the other chromosomes, rapidly evolving chromosome 2 is enriched in “unclassified proteins” (*P*-value 0.0). Very few pathogenicity factors have been found and annotated for *F. graminearum*, and many effector proteins are likely to belong to the unclassified proteins category. Indeed, we also find that chromosome 2 is highly enriched for in planta expressed genes. 31.3% of genes detected exclusively in planta in infected barley (Guldener et al. 2006) as well as 39% of genes detected exclusively in planta in infected wheat (Zhang et al. 2012) are located on chromosome 2, compared with 13.7–24.5% for the remaining three chromosomes. Furthermore, *F. graminearum* chromosome 2 noticeably shows more regions with high SNP density and recombination frequency than the other three chromosomes (fig. 2, track I). Taken together, this supports a more prominent role in virulence for genes on this particular chromosome*. Fusarium verticillioides* chromosomes 10 and 11 share macrosynteny with the interstitial region of *F. graminearum* chromosome 2. A MIPS FunCat functional enrichment analysis of *F. verticillioides* genes on chromosomes 10 and 11 supports the potential role in pathogenicity. Chromosome 10 is enriched in the functional category “disease, virulence, and defense” (*P*-value 0.04) and chromosome 11 in the category “secondary metabolism” (*P*-value 0.04), followed by “virulence, disease factors” (*P*-value 0.06).

From these observations we conclude that the dispensable chromosomes in *F. oxysporum* f. sp. *lycopersici* are rapidly evolving and that regions with higher numbers of rapidly evolving genes can be found on specific core chromosomes in *F. graminearum* and *F. verticillioides*. In particular *F. graminearum* chromosome 2 can be linked to potential roles in pathogenicity. To further understand genomic features involved in *Fusarium* pathogenicity, we investigate if particular groups of genes are under diversifying selection as described below.

### Diversifying Selection Acts Strongly on Genes Encoding [SG]-P-C-[KR]-P Proteins and Pathogen-Associated Proteins

In addition to chromosomal regions under diversifying selection, we inspected $d_{N}/d_{S}$ ratios and the percentage of genes under site-specific diversifying selection across specialized gene groups in *F. graminearum*, *F. oxysporum* f. sp. *lycopersici*, and *F. verticillioides* (table 2). We found that for the three genomes, site-specific diversifying selection acts most strikingly on genes encoding proteins with an N-terminal [SG]-P-C-[KR]-P sequence motif implicated in sensing of host signals (Zhang et al. 2012), followed by pathogen-associated proteins, which are defined as proteins that have sequence similarity hits specific to fungal pathogens and at the same time predominantly absent from nonpathogenic fungi (Sperschneider et al. 2013). For example, the percentage of *F. graminearum* genes that have sites under diversifying selection as predicted by CODEML rises to 14.7% for genes encoding proteins with an N-terminal [SG]-P-C-[KR]-P sequence motif and to 8.6% for genes encoding pathogen-associated proteins, compared with the genome-wide level of 1.6%. Significantly higher percentages of genes under site-specific diversifying selection are also found in secreted proteins for *F. graminearum*, but not for *F. verticillioides* and *F. oxysporum* f. sp. *lycopersici*. This is likely due to a lack of orthologies for *F. verticillioides* and *F. oxysporum* f. sp. *lycopersici* in the *Fusarium* genus that only allowed a small number of genes encoding secreted proteins to be analyzed using CODEML. In contrast, no enrichment in genes undergoing diversifying selection is predicted for the group of genes encoding carbohydrate-active enzymes and transcription factors. In general, carbohydrate-active enzymes can degrade, modify, or create glycosidic bonds and the majority of these can be expected to undergo purifying selection (Brunner et al. 2013). In contrast, proteins that are conserved predominantly across fungal pathogens are strong candidates for being involved in pathogenicity (Gene list given in supplementary material, Supplementary Material online) and this is reflected in diversifying selection pressure acting on this group. A MIPS FunCat functional enrichment analysis of genes under diversifying selection that encode pathogen-associated proteins for *F. graminearum* and *F. oxysporum* f. sp. *lycopersici* genes reveals an enrichment in unclassified proteins. The group of genes encoding proteins with an N-terminal [SG]-P-C-[KR]-P sequence motif also carry a secretion signal but have not been functionally characterized (Sperschneider et al. 2013). Thus, their role in adaptation remains elusive.

**Table 2:** Diversifying Selection Analysis for Three *Fusarium* Genomes across Different Protein Groups

	**No. of Genes Analyzed (YN00/CODEML)**			**Mean** $d_{N}/d_{S}$ **Ratio**			**% of Genes with Sites under Diversifying Selection**		
**Gene Classification**	***FG***	***FV***	***FO***	***FG***	***FV***	***FO***	***FG***	***FV***	***FO***
Genome-wide	11,863/11,501	13,638/12,826	16,434/14,575	0.15	0.15	0.2	1.6	2.1	2.9
Cazys	368/365	459/443	522/490	0.09	0.09	0.11	1.6	1.1	1.4
Transcription factors	548/541	622/607	742/703	0.13	0.13	0.18	0.7	1.2	1
Trichothecenes	12/11	—	—	0.2	—	—	0	—	—
Fumonisins	—	15/2	—	—	0.2	—	—	0	—
Secreted	1,270/1,246	1,398/1,322	1,539/1,434	0.16	0.16	0.19	2.4*	2.8	2.4
Secreted, small, cysteine-rich	313/304	325/291	372/338	0.2	0.2	0.23	3.9*	3.8	2.1
[SG]-P-C-[KR]-P sequence motif	35/34	43/37	38/37	0.4	0.43	0.41	14.7*	24.3*	21.6*
Pathogen-associated	1,065/880	—	2,371/1,966	0.39	—	0.38	8.5*	—	8.6*

Note.—$d_{N}/d_{S}$ ratios were calculated using YN00 for *F. graminearum* (*FG*), *F. verticillioides* (*FV*), and *F. oxysporum* f. sp. *lycopersici* (*FO*) genes with at least one ortholog. Diversifying selection acting on sites was calculated using CODEML for genes with at least two orthologs. These analyses were conducted on a genome-wide level as well as across certain protein categories. Carbohydrate-active enzymes are abbreviated as Cazys. Pathogen-associated proteins are given in Sperschneider et al. (2013) for *F. graminearum* (*FG*) and *F. oxysporum* f. sp. *lycopersici* (*FO*), but not *F. verticillioides* (*FV*). Statistical significance for each group compared with the remainder of the genome was assessed with a Fisher’s exact test at a significance threshold of *P* < 0.05 and is indicated with an asterisk (*).

*Fusarium graminearum* and *F. verticillioides* are producers of certain secondary metabolites called mycotoxins. In *F. graminearum*, the trichothecene mycotoxins contribute to virulence (Proctor et al. 1995) and *F. verticillioides* produces fumonisins encoded by a cluster of 15 genes (Proctor et al. 2003). These groups of mycotoxin genes are predicted to undergo purifying selection (table 2). In contrast, *F. oxysporum* f. sp. *lycopersici* is the only known *Fusarium* species that includes strains that interact with their host plant in a gene-for-gene relationship. *Fusarium oxysporum* f. sp. *lycopersici* secretes several proteins during infection of the host and these are referred to as SIX proteins (Houterman et al. 2007). We were able to analyze $d_{N}/d_{S}$ ratios for three of the SIX genes (*SIX1*, *SIX2*, *SIX9*) due to the presence of the gene in the genome sequence of the race two strain *F. oxysporum* 4287 and orthology to *F. oxysporum* 5176*.* All three *SIX* genes show elevated levels of $d_{N}/d_{S}$ ratios (*SIX1* 1.02, *SIX2* 0.31, *SIX9* 0.34). SIX1 is an effector that is recognized by a host *R*-gene and also referred to as Avr3 for its gene-for-gene relationship with the *I-3* tomato resistance gene (Rep et al. 2004) and shows diversifying selection when comparing *F. oxysporum* 4287 to *F. oxysporum* 5176. It is located on the dispensable chromosome 14, which is referred to as a pathogenicity chromosome and has been shown to turn a nonvirulent recipient strain virulent toward tomato via acquisition of the entire chromosome (Ma et al. 2010).

Taken together, the comparison of $d_{N}/d_{S}$ ratios strongly supports the existence of a *Fusarium* two-speed structure on the gene group level. All three *Fusarium* species have core groups of genes under purifying selection to preserve their function and specialized groups of genes evolving at a faster rate, such as those encoding proteins with an N-terminal [SG]-P-C-[KR]-P sequence motif and pathogen-associated proteins. The strong functional association of these rapidly evolving gene groups with pathogenicity suggests that the signal of adaptation in *Fusarium* can point toward effector genes that need to adapt to host defense responses.

### Genes with $d_{N}/d_{S}$ > 1 and a Predicted Secretion Signal Are Found in *F. oxysporum* f. sp. *lycopersici* and *F. verticillioides*, but Not in *F. graminearum*

Pathogens are under strong pressure to adapt to their hosts and the evolution of a successful pathogen is influenced by the ability of the host to evolve resistance. Pathogen genes that need to adapt to the host and its defense mechanisms or that need to avoid host recognition can be expected to undergo diversifying selection. We initially examined *F. graminearum*, *F. oxysporum* f. sp. *lycopersici*, and *F. verticillioides* genes with $d_{N}/d_{S}$ ratios > 1 and a secretion signal predicted by SignalP 4.1 (Petersen et al. 2011) due to their likely role as effectors in host infection. We found that 60 *F. graminearum* genes, 48 *F. verticillioides* genes, and 257 *F. oxysporum* f. sp. *lycopersici* genes have $d_{N}/d_{S}$ ratio > 1, which is a likely indication of diversifying selection pressure (table 3). None of the 60 *F. graminearum* genes has a predicted secretion signal, whereas 3 *F. verticillioides* genes and 10 *F. oxysporum* f. sp. *lycopersici* are predicted to be secreted (table 4). The majority (83%) of *F. graminearum* genes with $d_{N}/d_{S}$ ratio > 1 belong to the pathogen-associated category. For *F. verticillioides* and *F. oxysporum* f. sp. *lycopersici*, the majority of genes with $d_{N}/d_{S}$ ratio > 1 are unclassified. We speculate that the lack of genes with $d_{N}/d_{S}$ ratios > 1 and a secretion signal in *F. graminearum* can be explained as follows. *Fusarium graminearum* might be secreting proteins in early stages of infection that are under diversifying selection due to direct interaction with its plant host, however the underlying genes do not have orthologs in the *Fusarium* genus or the selection signal could be more subtle than $d_{N}/d_{S}$ > 1. Using $d_{N}/d_{S}$ > 1 to identify diversifying selection is known to lack power as adaptation might be acting only on a small number of sites in a protein or only on certain branches in the lineage. Therefore, in the following we additionally use site-specific diversifying selection predicted by CODEML to identify genes that have undergone adaptation.

**Table 3:** A Comparison of Genes with $d_{N}/d_{S}$ > 1 for Three *Fusarium* Genomes across Different Protein Groups

	**No. of Genes with** $d_{N}/d_{S}$ **Ratio > 1**		
**Gene Classification**	***FG***	***FV***	***FO***
Genome-wide	60	48	257
Cazys	1	0	0
Transcription factors	0	0	5
Trichothecenes	0	—	—
Fumonisins	—	0	—
Secreted	0	3	10
Secreted, small, cysteine-rich	0	0	3
N-terminal [SG]-P-C-[KR]-P sequence motif	0	2	0
Pathogen-associated	50	—	99

Note.—The number of genes with $d_{N}/d_{S}$ > 1 across different protein groups is given for *F. graminearum* (*FG*), *F. verticillioides* (*FV*), and *F. oxysporum* f. sp. *lycopersici* (*FO*). Carbohydrate-active enzymes are abbreviated as Cazys.

**Table 4:** Genes in *F. oxysporum* f. sp. *lycopersici* and *F. verticillioides* that Have $d_{N}/d_{S}$ Ratios > 1 and Are Predicted to be Secreted by SignalP 4.1

**Protein ID**	**Length (amino acids)**	**Cys**	**Pfam Domain Hits**	**Chromosome**	**[SG]-P-C-[KR]-P Motif**	$d_{N}/d_{S}$
FOXG_10753	305	2	—	7	—	1.17
FOXG_12439	162	—	—	3	—	1.69
FOXG_13567	196	—	—	12	—	1.68
FOXG_14098	141	—	—	6	—	1.69
FOXG_16418 (SIX1, *Avr3*)	284	8	—	14	—	1.02
FOXG_17395	163	6	—	—	—	1.15
FOXG_17509	342	5	Fungal specific transcription factor domain (1.8e-15)	—	—	1.36
FOXG_19078	104	—	—	2	—	1.48
FOXG_19890	67	1	—	2	—	1.79
FOXG_22856	208	8	—	6	—	1.01
FVEG_15341	546	17	—	—	GPCKP	1.11
FVEG_17224	92	1	—	—	—	1.24
FVEG_17416	278	2	—	—	GPCRP	1.28

Note.—Note that no genes with $d_{N}/d_{S}$ ratios > 1 and a predicted secretion signal were found for *F. graminearum.* Cys stands for the number of cysteines in the sequence.

### Fusarium graminearum Genes Potentially Involved in Host Adaptation Include Three Cell Wall Degrading Enzymes

Effector candidates that are potentially involved in host adaptation or evasion were identified as follows: they are 1) under diversifying selection, 2) differentially expressed during infection, and 3) predicted to be secreted. As a number of experimentally verified fungal effectors have been found to be of larger size and not rich in cysteines, we did not included these criteria for effector finding here (Sperschneider et al. 2015). In particular, we collected the set of *F. graminearum* genes that are detected as differentially expressed in planta in at least one of several experiments (see Materials and Methods) and are under diversifying selection, that is, they pass the CODEML LRTs if they have at least two orthologs or they have a $d_{N}/d_{S}$ ratio larger than the genome-wide mean if they only have one ortholog. This resulted in 128 genes and 29 of these have a predicted signal peptide using SignalP 4.1 (Petersen et al. 2011). We propose that these 29 genes are likely to be involved in host adaptation or evasion and are thus strong effector candidates (table 5).

**Table 5:** The List of 29 *F. graminearum* Proteins that Are Expressed during in planta Conditions, Are under Diversifying Selection and Have a Predicted Signal Peptide Using SignalP 4.1 (Petersen et al. 2011)

**ID**	**Length**	**[SG]-P-C-[KR]-P Motif**	**Functional Annotation (MIPS)**	**Phyre2 Structure Prediction (confidence/coverage)**	**In planta Barley**	**In planta Wheat**	**16HAI**	**40HAI**	**64HAI**	**Pathogen- Associated**
FGSG_07625	325	—	Probable alpha-L-arabinofuranosidase precursor	Alpha-l-arabinofuranosidase (100%/92%)	Yes	—	Yes	Yes	Yes	—
FGSG_10561	1480	—	Related to RF2 protein	—	—	—	—	Yes	Yes	—
FGSG_10999	231	—	xylA	Gh11 xylanase (100%/90%)	Yes	—	Yes	—	Yes	—
FGSG_04521	265	GPCKP	—	—	—	—	Yes	Yes	—	Yes
FGSG_04841	273	GPCKP	—	Endoglucanase (99.3%/54%)	—	—	Yes	—	—	Yes
FGSG_05947	281	GKCKP	—	—	—	—	Yes	Yes	—	Yes
FGSG_07629	278	GPCKP	—	Endo-1,4-beta-xylanase y (99.5%/53%)	—	—	Yes	Yes	—	Yes
FGSG_07684	238	GPCRP	—	Wnt inhibitory factor 1 (99.2%/36%)	—	—	Yes	—	—	—
FGSG_12405	182	GPCRP	—	—	—	—	Yes	Yes	—	Yes
FGSG_15977	295	GLCKP	—	—	—		Yes	—	—	—
FGSG_02255	221	—		ECP6 (99.1%/32%)	—	—	Yes	—	—	Yes
FGSG_03550	186	—	Related to early nodulin 75 precursor	—	—	—	Yes	—	—	Yes
FGSG_04597	224	—		Galactose-binding domain-like (99.9%/72%)	—	—	Yes	—	—	Yes
FGSG_03315	411	—	Related to endopeptidase K	Subtilisin-like (100%/87%)	—	Yes	—	Yes	Yes	—
FGSG_13955	435	—		—	—	Yes	Yes	—	Yes	—
FGSG_15136	69	—		—	—	Yes	Yes	Yes	Yes	—
FGSG_15175	91	—		—	—	Yes	—	—	Yes	—
FGSG_03958	400	—		—	Yes	—	—	—	—	—
FGSG_16163	513	—		Homoserine o-acetylytransferase (99.9%/68%)	Yes	—	—	—	—	—
FGSG_01588	724	—	—	—	—	—	Yes	Yes	—	Yes
FGSG_12081	139	—		—	—	—	Yes	—	Yes	—
FGSG_03969	501	—		Low density lipoprotein receptor variant (98.6%/81%)	—	—	Yes	—	Yes	—
FGSG_01594	163	—		—	—	—	—	Yes	Yes	—
FGSG_10560	251	—		—	—	—	—	Yes	Yes	—
FGSG_10562	286	—		—	—	—	—	Yes	Yes	—
FGSG_01822	268	—		Ephrin type-a receptor 2 (95.5%/15%)	—	—	—	—	Yes	—
FGSG_08245	634	—		—	—	—	—	—	Yes	—
FGSG_08196	260	—	Related to aspergillopepsin II precursor	Peptidase A4 (100%/75%)	—	—	—	Yes	—	—
FGSG_17084	373	—		—	—	—	Yes	Yes	Yes	—

NOTE.—Two *in planta* experiments were used, infected barley (Guldener et al. 2006) and infection of wheat as well as specific time points (16 HAI, 40 HAI, and 64HAI) of that experiment (Zhang et al. 2012). Pathogen-associated refers to proteins that have sequence similarity hits specific to fungal pathogens and at the same time predominantly absent from nonpathogenic fungi (Sperschneider et al. 2013).

Four of our candidates (table 5) are preferentially expressed during invasive growth in planta upon infection of wheat (Zhang et al. 2012), that is, *FGSG_03315*, *FGSG_13955*, *FGSG_15136*, and *FGSG_15175*. Using phmmer searches against the UniProt database we find that *FGSG_13955* is found exclusively in *F. graminearum* and *Verticillium*. FGSG_15136 has sequence similarity only to the two cereal pathogens *F. pseudograminearum* and *F. culmorum*. Homology to FGSG_15175 is found in different *Fusarium* species as well as in *Verticillium*. Using differential expression data from Zhang et al. (2012), six genes (*FGSG_02255*, *FGSG_03550*, *FGSG_04597*, *FGSG_04841*, *FGSG_07684*, *FGSG_15977*) are differentially expressed exclusively at the early time point of 16 h after inoculation, termed covert penetration (Zhang et al. 2012). FGSG_02255 contains a LysM domain and has a predicted structure similar to the Ecp6 effector (de Jonge et al. 2010) using Phyre2 (Kelley and Sternberg 2009). *FGSG_03550* is located in close proximity to the trichothecene gene cluster. Strikingly, FGSG_15977 shares significant sequence similarity only with the cereal pathogen *Fusarium pseudograminearum*, *Fusarium culmorum* as well as the wheat pathogen *S. nodorum* and the strawberry infecting *Colletotrichum gloeosporioides* (strain Nara gc5). FGSG_04841 and FGSG_07684 are [SG]-P-C-[KR]-P sequence motif proteins. FGSG_15977 also features a modified version of the [SG]-P-C-[KR]-P motif after its signal peptide (G-L-C-K-P).

Three of the genes in table 5 are annotated as encoding cell wall degrading enzymes, that is, FGSG_07625, FGSG_10561, and FGSG_10999. FGSG_07625 is predicted to belong to the GH62 α-l-arabinofuranosidase family. These enzymes remove the α-l-arabinosyl substituents from arabinoxylan, which is a hemicellulose found in plant cell walls, in particular cereal grains. FGSG_10561 is a long protein that has been classified by the MIPS FunCat catalogue as a toxin with chitinase activity. It contains a LysM domain (PF01476, 2.3e-06), a Glycosyl hydrolases family 18 domain (PF00704, 6.6e-48) as well as the putative necrosis-inducing factor domain (PF14856, 2e-24) from the Ecp2 effector protein from the tomato pathogen *Cladosporium fulvum*. The two neighboring genes *FGSG_10560* and *FGSG_10562*, of unknown function, are also expressed in planta, are under diversifying selection and have a predicted signal peptide.

**Fig. 4.—:**
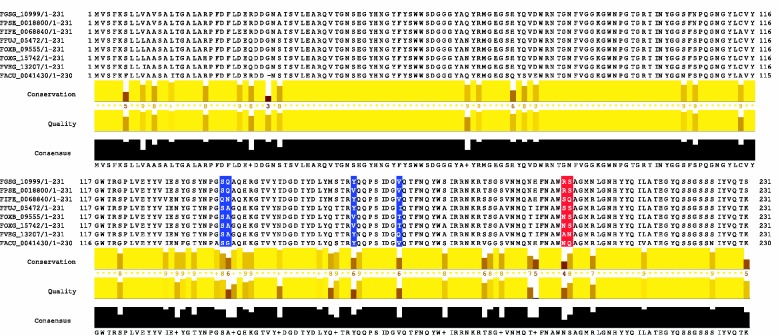
*XylA* shows signatures of site-specific diversifying selection. An alignment of XylA (FGSG_10999) protein sequences in the *Fusarium* genus reveals a high conservation on the amino acid level, visualized by Jalview (Waterhouse et al. 2009). However, two sites (shown as red columns) are predicted to undergo diversifying selection by CODEML (model M7/M8). Additionally, four sites (shown as blue columns) have a posterior probability larger than 0.5 as estimated using the CODEML model M7/M8 empirical Bayes method, including Val151 (shown at position 162 in this alignment). *F. graminearum*: FGSG, *F. pseudograminearum*: FPSE, *F. culmorum*: FCUL, *F. incarnatum–F. equiseti*: FIFE, *F. fujikuroi*: FFUJ, *F. oxysporum* Fo5176: FOXB, *F. oxysporum* f. sp. *lycopersici*: FOXG, *F. verticillioides*: FVEG, *F. acuminatum*: FACU.

FGSG_10999 is a family 11 endo-beta-1,4-xylanase, also referred to as XylA. Xylan is a significant component especially in the cell wall of cereal plants and xylan-degrading enzymes are thought to play a role in pathogen attack, particularly of monocots (Belien et al. 2006). Functional studies of microbial endoxylanases and their plant inhibitors suggest a coevolutionary process in which plants evolve proteinaceous inhibitors of endoxylanases to combat microbial attack while microbial pathogens evolve endoxylanases with distinct sensitivities toward the respective plant inhibitors (Belien et al. 2005). XylA in particular has been shown to be inhibited by the *Triticum aestivum* xylanase inhibitor TAXI-I, but unexpectedly not by the xylanase inhibitor protein XIP-I (Belien et al. 2005). Insensitivity of XylA to XIP-1 has been attributed a handful of amino acid changes in XylA, in particular Val151 (Belien et al. 2007). CODEML returns two sites under diversifying selection (fig. 4). Furthermore, we do not detect significant site-specific diversifying selection for XylB (FGSG_03624), which is a necrotizing factor but is not essential for virulence (Sella et al. 2013). This emphasizes the diversification of endoxylanases and that subtle amino acid changes can be a driving force in the coevolutionary arms race between plant and pathogen.

In summary, the discovery of *F. graminearum* genes that are predicted to be secreted, rapidly evolving and expressed in early stages of infection strongly links them to effector function. In particular, it points toward a specialized role of particular cell wall degrading enzymes in host adaptation or evasion. This supports recent findings of plant cell wall degrading enzymes in a hemibiotroph that are likely affected by or driving coevolution of host and pathogen (Brunner et al. 2013). In addition, several [SG]-P-C-[KR]-P sequence motif proteins appear as candidates possibly involved in host adaptation. The [SG]-P-C-[KR]-P sequence motif proteins are a diverse group without a characterized function and will be explored further in the next section.

### [SG]-P-C-[KR]-P Proteins Consist of Diverse C-Terminal Domains with Variable Evolutionary Pressure

The [SG]-P-C-[KR]-P sequence motif immediately after a signal peptide is specific to *Fusarium* proteins (Sperschneider et al. 2013), however its function remains elusive. We collected proteins from *F. graminearum*, *F. verticillioides*, and *F. oxysporum* f. sp. *lycopersici* that have a [SG]-P-C-[KR]-P sequence motif in the first 50 amino acids. These 118 proteins share a common architecture composed of a predicted signal peptide, a [SG]-P-C-[KR]-P sequence motif followed by a serine/threonine-rich region of variable size and a variable C-terminus (Sperschneider et al. 2013). The [SG]-P-C-[KR]-P sequence motif has thus far not been functionally characterized. The presence of a serine/threonine-rich region is likely to play a role in *O*-glycosylation and hints toward a role related to the fungal cell wall. Zhang et al. (2012) report transient induction at 16 h after inoculation during wheat coleoptile infection and at 2–8 h during in vitro germination for these genes and suggested a role as cell wall-anchored proteins in sensing of host signals.

**Fig. 5.—:**
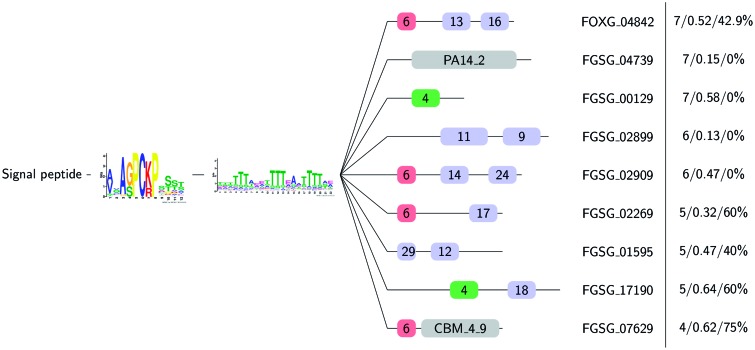
Examples for the variable C-terminal domain structures found in the [SG]-P-C-[KR]-P proteins. The [SG]-P-C-[KR]-P proteins feature a predicted signal peptide followed by the [SG]-P-C-[KR]-P motif, a serine/threonine rich stretch of variable length and a C-terminal domain. A representative member of the predicted tribes is shown on the right. The number of tribe members is given in the far right column followed by the mean $d_{N}/d_{S}$ ratio and the percentage of tribe members that are under site-specific diversifying selection predicted by CODEML. Meme motifs are given with their respective number (see supplementary material, Supplementary Material online, for motifs) and Pfam domain hits are given with their identifier. PA14_2 (Pfam PF10528) is a GLEYA domain found in fungal adhesins and CBM_4_9 (Pfam PF02018) is found in carbohydrate-active enzymes.

The C-terminal region for the [SG]-P-C-[KR]-P proteins is composed of a variety of different domain structures, which indicates the functional diversity and potential specialization of members in this group. We collected the C-terminal protein sequences and clustered them into families using TRIBE-MCL. This resulted in 25 predicted tribes covering 113 of the 118 genes. We were able to obtain $d_{N}/d_{S}$ ratios for 116 genes and to calculate the likelihood of site-specific diversifying selection for 108 genes. The percentage of genes that are under site-specific diversifying selection (20.2%) is substantially higher than genome-wide means (table 1). We ran MEME and Pfam searches to inspect the variable C-terminal domain structures. Figure 5 shows examples of the diverse domain structures for the [SG]-P-C-[KR]-P proteins with a representative member (see supplementary material, Supplementary Material online, for full results). One tribe (FGSG_04739, FGSG_04858, FVEG_05717, FVEG_07656, FVEG_11011, and FOXG_13582, FOXG_02535) shares sequence similarity in the C-terminal region with the GLEYA domain (PA14_2, Pfam PF10528) found in fungal adhesins. The mean $d_{N}/d_{S}$ ratio for this tribe is 0.15, which is not elevated compared with the genome-wide means and therefore suggests a conserved function. In contrast, another tribe (FGSG_07629, FVEG_03625, FOXG_01333, and FOXG_05752) contains a carbohydrate binding domain (CBM_4_9, Pfam PF02018) found in carbohydrate-active enzymes. This tribe has an elevated $d_{N}/d_{S}$ ratio of 0.62 and FGSG_07629, FVEG_03625, and FOXG_05752 show site-specific diversifying selection on amino acids in the C-terminal region. Taken together, this exemplifies the diversification of the [SG]-P-C-[KR]-P proteins through their C-terminals or a potential acquisition of the N-terminal SG]-P-C-[KR]-P motif through convergent evolution.

## Discussion

Pathogens can be expected to harbor conserved pathogenicity genes as well as dynamic pathogenicity genes that need to keep up with the host’s ability to defend pathogen attacks. Many conserved *Fusarium* pathogenicity genes have been characterized (Ma et al. 2013), whereas those linked to host adaptation or host evasion have remained largely undefined, apart from the set of SIX effectors in the tomato-infecting *F. oxysporum* f. sp. *lycopersici* (Houterman et al. 2007). The evolution of pathogenicity and host specificity in *Fusarium* has involved multiple and varied processes of genome innovation, for example, population diversity is found in specific genomic regions (Cuomo et al. 2007) while the horizontal acquisition of pathogenicity-related genes (Gardiner et al. 2012) as well as whole pathogenicity-related chromosomes occurred (Ma et al. 2010). However, the impact of diversifying selection on *Fusarium* pathogen genomes, gene groups, and individual genes involved in host–pathogen interactions remains unexplored. In this work, we investigated diversifying selection in three *Fusarium* pathogens that have over time evolved distinct pathogenicity profiles. We showed that adaptive evolution generates a two-speed genome structure for *F. graminearum*, *F. verticillioides*, and *F. oxysporum* f. sp. *lycopersici*, both on the chromosomal and the gene group level, and that the signal of adaptation combined with in planta expression data can be used to identify individual genes involved in *Fusarium*-host interactions.

Diversifying selection acts strongly on the dispensable chromosomes in *F. oxysporum* f. sp. *lycopersici*, but it is also present in genomes without dispensable chromosomes. Highly polymorphic regions in *F. graminearum* were previously found at chromosome ends as well as in large interstitial regions of chromosomes 1, 2, and 4 (Cuomo et al. 2007). However, population diversity using strain information captures different evolutionary processes than diversifying selection in the genus. For *F. graminearum*, we find that regions containing genes under site-specific diversifying selection mostly overlap with regions of population diversity (Cuomo et al. 2007). However, we were able to additionally identify rapidly evolving genes potentially involved in ancient niche or host specialization in the interstitial region of chromosome 3. Furthermore, *F. graminearum* chromosome 2 was predicted as a hotspot for rapid evolution and in planta expressed genes. We anticipate that the dispensable chromosome 12 that has been found in the *F. verticillioides* genetic map (Xu and Leslie 1996), but not in the genome assembly (Ma et al. 2010), may also be rapidly evolving.

The enrichment of *F. graminearum* chromosome 2 in genes expressed in planta infection of wheat and barley (Guldener et al. 2006; Zhang et al. 2012) might point to a prominent role in infection of cereals. Furthermore, the interstitial region of *F. graminearum* chromosome 2 shares macrosynteny with *F. verticillioides* chromosomes 10 and 11, which are also enriched in genes annotated as virulence factors. Apart from genomic hotspots for adaptation, we found significantly higher percentage of genes under site-specific diversifying selection in groups encoding [SG]-P-C-[KR]-P sequence motif proteins and in pathogen-associated proteins for all three *Fusarium* species. Interestingly, genes encoding [SG]-P-C-[KR]-P sequence motif proteins have diverse C-terminal domain structures with variable signatures of diversifying selection. Together with their likely location in the fungal cell wall this could point toward a role in sensing of host signals or potential host adaptation and associated pathogen coevolution.

After revealing evolutionary dynamic *Fusarium* chromosomes and gene groups, we investigated the impact of site-specific diversifying selection on *F. graminearum* genes that are in planta expressed to elucidate genes potentially involved in the early biotrophic infection phase. In particular, we found 29 *F. graminearum* genes that were expressed at an early time point in wheat infection called covert penetration (Zhang et al. 2012) and that are at the same time rapidly evolving and predicted to be secreted, which strongly indicates an effector function. It is important to reiterate that we were only able to calculate signatures of diversifying selection for 83.2% of genes in *F. graminearum*, and thus there will be effectors that we will miss in our analysis. Nevertheless, this study points towards a specialized role of three cell wall degrading enzymes in host adaptation or host evasion, including the *XylA* gene that is known to be involved in the coevolutionary arms race (Belien et al. 2006). Similarly, six cell wall degrading enzymes were found to be involved in either host adaptation or host evasion in the wheat-infecting hemibiotroph *Z. tritici* (Brunner et al. 2013).

Our results have demonstrated that diversifying selection has a strong impact not only on biotrophic pathogens with an intimate connection to their hosts, but also on hemibiotrophs with an extended necrotrophic phase such as *Fusarium* pathogens. Our work provides a valuable resource for researchers studying the impact of adaptation in pathogen–host coevolution as well as a focussed set of *Fusarium* gene targets for functional knockout studies. We showed that combined with in planta expression data, diversifying selection is a powerful signal for predicting pathogenicity genes involved in the arms race between host and pathogen. Future improvements will come in the form of improved gene annotations, a greater sequencing depth in the genus for picking up weaker signals of diversifying selection as well as the generation of in planta expression data sets for other *Fusarium* species that capture early time points during infection reliably for effector prediction.

## Supplementary Material

Supplementary material is available at *Genome Biology and Evolution* online (http://www.gbe.oxfordjournals.org/).
